# Liver MRI and clinical findings to predict response after drug eluting bead transarterial chemoembolization in hepatocellular carcinoma

**DOI:** 10.1038/s41598-021-01839-6

**Published:** 2021-12-15

**Authors:** Jeong Yeop Lee, Byung Chan Lee, Hyoung Ook Kim, Suk Hee Heo, Sang Soo Shin, Yong Yeon Jeong

**Affiliations:** 1 Department of Radiology, Chonnam National University Hwasun Hospital, 322 Seoyang-ro, Hwasun, 58128 Korea; 2 Department of Radiology, Chonnam National University Hospital, 42 Jebong-ro, Dong-gu, Gwangju, 61469 Korea; 3 Department of Radiology, Chonnam National University Medical School, 42 Jebong-ro, Dong-gu, Gwangju, 61469 Korea

**Keywords:** Predictive markers, Hepatology, Liver cancer

## Abstract

To identify the gadoxetic acid (GA)-enhanced magnetic resonance imaging (MRI) and laboratory findings that enable prediction of treatment response and disease-free survival (DFS) after the first session of drug eluting bead transarterial chemoembolization (DEB-TACE) in patients with hepatocellular carcinoma (HCC). A total of 55 patients who underwent GA-enhanced MRI and DEB-TACE from January 2014 to December 2018 were included. All MRI features were reviewed by two radiologists. Treatment response was evaluated according to the modified Response Evaluation Criteria in Solid Tumors. Univariate and multivariate logistic regression analyses were used to determine predictive factors of treatment response and DFS, respectively. A total of 27 patients (49.1%) achieved complete response (CR) after one session of treatment. There were no significant differences between the two groups in terms of clinical and laboratory characteristics. Heterogeneous signal intensity in the hepatobiliary phase (HBP) was the only independent predictor of non-CR (odds ratio, 4.807; *p* = 0.048). Recurrent HCC was detected in 19 patients (70.4%) after CR. In the multivariate analysis, elevated serum alpha-fetoprotein (AFP) level (≥ 30 ng/mL) was the only significant parameter associated with DFS (hazard ratio, 2.916; *p* = 0.040). This preliminary study demonstrated that heterogeneous signal intensity in the HBP and high serum AFP were useful predictive factors for poor treatment response and short DFS after DEB-TACE, respectively.

## Introduction

Hepatocellular carcinoma (HCC) is the sixth most common type of cancer and was the fourth leading cause of cancer-related death worldwide in 2018^1^. Treatment options for HCC depend on the stage at the time of diagnosis. Surgical resection, liver transplantation, and radiofrequency ablation (RFA) are curative treatments for HCC. However, these therapeutic options are only appropriate for very early or early stage HCC^2–4^, and only about 30–40% of patients are diagnosed in these stages^5^. Transarterial chemoembolization (TACE) is a secondary procedure that can be effective for cases in which curative treatment is not an option, and has been established as the standard treatment for intermediate stage HCC^2^. TACE is also used for slowing the progression of cancer in patients waiting for liver transplantation^6^. Moreover, it can be an alternative therapeutic option when RFA is not possible due to the location of the lesion or when surgery is restricted due to other underlying diseases^7^.

TACE with drug eluting beads (DEB-TACE) is a subtype of TACE^8^. For this technique, chemotherapeutic agents are contained in a microsphere and gradually released within the tumor. This process allows greater and prolonged drug retention in HCC and lower release of the drug into the systemic circulation. Several studies have reported that peak serum concentrations of doxorubicin are significantly lower after DEB-TACE than after conventional TACE^8,9^, resulting in fewer systemic complications^9,10^. DEB-TACE is currently being used in practice, but two large prospective randomized trials have not proven supremacy of DEB-TACE over conventional TACE in the treatment of HCC^10,11^.

Follow-up computed tomography (CT) and magnetic resonance imaging (MRI) after TACE are useful for diagnosing and evaluating the response. Gadoxetic acid (GA)-enhanced liver MRI has been widely used to evaluate HCC due to its excellent diagnostic performance. For patients with HCC, prognosis is strongly associated with the degree of response to the first TACE^12,13^. Therefore, predicting the response to TACE based on pretreatment MRIs could be used for establishing a treatment strategy. Recently, several studies have investigated pretreatment imaging findings, including MRIs, which may be predictors of treatment response or overall outcomes in conventional TACE^14–18^. However, there are fewer studies about DEB-TACE than conventional TACE. Some studies have mentioned the MRI findings in DEB-TACE, but they focused on angiographic characteristics rather than specific MRI findings^19,20^ or evaluated only posttreatment MRI^21^. Our study aimed to identify pretreatment GA-enhanced MRI findings that may enable the prediction of treatment response and disease-free survival (DFS) after the first session of DEB-TACE in patients with HCC.

## Methods

### Patients

**Figure 1:**
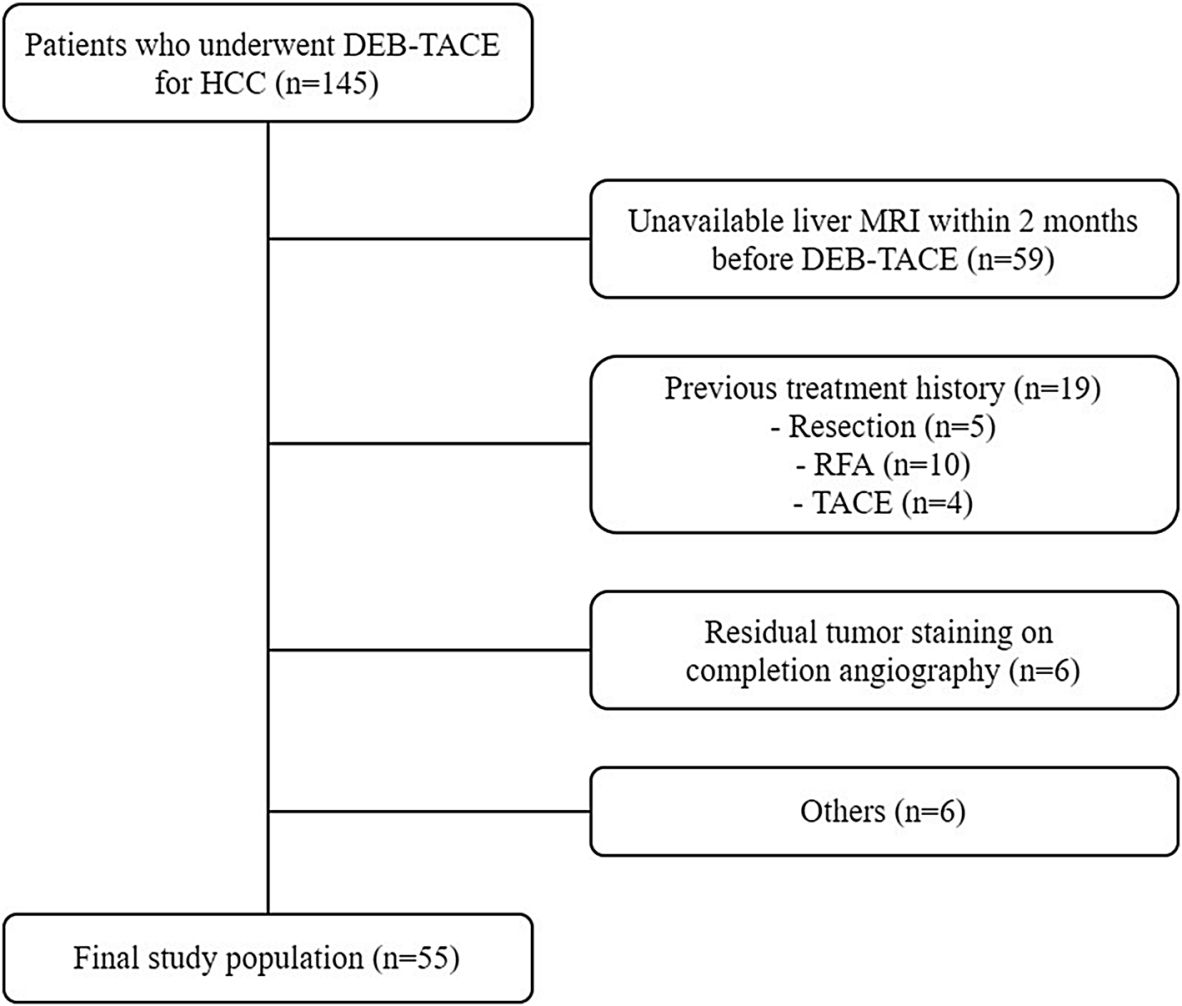
Flow chart of the study population. *DEB-TACE* drug eluting bead transarterial chemoembolization, *HCC* hepatocellular carcinoma, *MRI* magnetic resonance imaging, *RFA* radiofrequency ablation.

We retrospectively analyzed the patient database at a single tertiary hospital (Chonnam National University Hwasun Hospital, Hwasun, Korea). The Institutional Review Board of Chonnam National University Hwasun Hospital approved this study and waived informed consent requirement due to the retrospective study design. All methods were carried out in accordance with relevant guidelines and regulations. From January 2014 to December 2018, 145 consecutive patients underwent DEB-TACE for HCC at our institution. HCC diagnosis was based on imaging following the American Association for the Study of Liver Diseases (AASLD) criteria^3^. In our study, MRI was used for all HCC diagnoses. The exclusion criteria for our study were as follows: (a) no available GA-enhancement liver MRI within 2 months of DEB-TACE; (b) history of previous treatment for HCC, including surgical resection, RFA, TACE, or other therapies; (c) residual tumor staining on completion angiography during TACE; (d) other concurrent malignancies; and (e) insufficient follow-up examination. Ultimately, 55 patients were enrolled in the study (Fig. 1).

The following clinical information, laboratory and procedural factors that could have potentially influenced the therapeutic response were analyzed: age, sex, etiology of liver disease, presence of liver cirrhosis, Child–Pugh class, serum alpha-fetoprotein (AFP), protein induced by vitamin K absence or antagonist-II (PIVKA-II), and DEB particles size.

### MRI techniques

A 3T MR scanner (MAGNETOM Skyra; Siemens Healthineers) with an 18-channel body array coil was used for all imaging according to the specified protocols (Table 1). Breath-hold axial in- and opposed-phase T1-weighted images (T1WI) were obtained using the two-dimensional volumetric interpolated breath-hold examination (VIBE) technique. Breath-hold T2-weighted images (T2WI) were obtained using the fat-suppressed half-Fourier acquisition single-shot turbo-spin echo (HASTE) technique, and heavily T2WI was obtained using the breath-hold HASTE sequence without fat suppression. For contrast-enhanced dynamic T1WI using the 3D VIBE technique, 0.1 mL/kg (0.25 mmol/mL) of GA (Primovist®, Bayer Healthcare) was injected with an MRI-compatible injector (Nemoto, Kyorindo) at a flow rate of 1 mL/sec and followed by 20 mL of 0.9% saline flush. Axial images were acquired at 30, 60, 120, 180, 600, and 900 s after injection of the contrast agent, and imaging at 900 s (15 min) was considered the hepatobiliary phase (HBP). Additionally, contrast-enhanced coronal images were obtained at 5 min.

**Table 1:** Imaging parameters for the MRI protocols.

	In- & opposed-phase T1WI	T2WI & heavily T2WI	Dynamic contrast enhancement
Pulse sequence	VIBE	HASTE	VIBE
TR/TE (ms)	4.4/1.4, 2.3	2000/81,166	3.5/1.3
Flip angle (°)	9	90–150	13
Section thickness (mm)	3	5	3
Interslice gap (mm)	0	0.5	0
Field of view (mm)	300–400	300–400	300–400
Matrix number	320 × 256	320 × 256	352 × 194

*HASTE* half-Fourier acquired single-shot turbo spin echo, *T1WI* T1-weighted image, *T2WI* T2-weighted image, *TE* echo time, *TR* repetition time, *VIBE* volumetric interpolated breath-hold examination.

### Pretreatment MRI analysis

All GA-enhanced MR images were reviewed by two abdominal radiologists (with 25 and 15 years of experience) who were blinded to clinical information and pathological analysis. After the first independent image evaluation, any discrepancies in the results between the two reviewers were resolved by consensus. Firstly, the size, multiplicity, and location of the tumors were estimated. The tumor location was classified into two categories: peripheral or central. Central location was defined as a tumor located within 0.5 cm of the first or second branches of the portal vein, or a tumor located at least 3 cm away from the liver capsule^14^. In the case of multiple tumors, only when all tumors were not in the central location, it was regarded as a peripheral tumor location. In our study, all tumors had a nodular appearance and there was no infiltrative type HCC.

The following MR imaging features in each HCC were evaluated: most of these imaging features have been developed based on previous studies^22,23^. In patients with multiple HCC lesions, the largest tumor was selected for assessment.

- Signal heterogeneity in the arterial phase; a nonenhanced area within the tumor in the arterial phase^15^
- Arterial rim enhancement; the presence of irregular ring-like areas of enhancement with a central hypovascular area in the arterial phase
- Arterial peritumoral enhancement; a detectable portion of polygonal or crescent-shaped enhancement outside of the tumor border that became isointense with background liver parenchyma in the later dynamic phase
- Radiologic capsule; a distinct peripheral rim with delayed contrast enhancement involving more than 90% of the tumor circumference
- Marginal irregularity; a non-smooth margin with a budding portion at the periphery of the tumor protruding into the hepatic parenchyma in the HBP
- Peritumoral hypointensity in the HBP; a wedge-shaped or flame-like area of low signal intensity in the liver parenchyma located outside of the tumor border in the HBP
- Signal heterogeneity in the HBP; a iso- or hyperintense portion was present inside the tumor in the HBP, compared with the background liver^24^
- Grossly visible vascular invasion
- Conspicuous rim; a smooth and discernable peripheral rim of the tumor appearing hypointense or hyperintense on T1WI or T2WI
- Peritumoral hyperintensity on T2WI; a polygonal or wedge-shaped area of high signal intensity outside of the tumor border on T2WI
- Intratumoral fat; an intratumoral area with decreased signal intensity on opposed-phase T1WI compared with in-phase images
- Intratumoral hyperintense portion on T1WI

### Chemoembolization protocols

Doxorubicin (Adriamycin, Ildong) and DEB (DC Bead®, Biocompatibles UK) agents were used for the DEB-TACE technique. The procedures were carried out with DEB particles of different sizes ranging from 70–150 μm to 300–500 μm, according to the manufacturer’s instructions. The determination of the sizes of DEB particles used in procedures was made by interventional radiologists considering the tumor size. One vial of DEB agent was loaded with 50 mg of doxorubicin solution, and the preparation was suspended in 30 mL non-ionic iodized contrast agent (Xenetix®, Guerbet). The DEB suspension was administered slowly at a rate of approximately 1 mL/min, to avoid reflux and non-target embolization. The total dosage used for the TACE procedure was determined by the interventional radiologist based on the extent of the tumor burden, but the maximum dose per patient was 100 mg of doxorubicin. The goal of embolization was the stasis of arterial blood flow to the tumor. When multiple lesions were present, the same procedure was used for all lesions. If tumor staining remained on completion angiography due to the large size of the tumor, it was excluded from the study.

### Follow-up and response evaluation

The initial post-TACE images were obtained using a 64-multidetector CT scanner (Somatom Sensation 64, Siemens Healthineers; and LightSpeed VCT, GE Healthcare) 4 weeks after the TACE. All examinations were analyzed on site and a secondary reading for response evaluation was performed specifically for this study by an abdominal radiologist who was blinded to all clinical information except that all patients had undergone DEB-TACE. Follow-up contrast-enhanced CTs were repeated every 2–3 months.

The tumor response was estimated using the modified Response Evaluation Criteria in Solid Tumors (mRECIST)^25^. For mRECIST, response is based on residual arterial enhancement rather than pure tumor shrinkage measured by the greatest diameter of the lesion. There are four categories of tumor response according to mRECIST: complete response (CR), disappearance of any intratumoral arterial enhancement in all target lesions; partial response (PR), at least a 30% decrease in the sum of the diameters of the lesions showing arterial enhancement, taking as reference the baseline sum of the diameters of the target lesions; stable disease (SD), any cases that do not qualify for either PR or progressive disease (PD); and PD, at least a 20% increase in the sum of the diameters of the lesions showing arterial enhancement, taking as reference the smallest sum of the diameters of the target lesions. For our study, all patients were categorized either into the CR or the non-CR group. The non-CR group included patients with PR or SD; there were no cases of PD in this study. Tumor recurrence after CR was defined as newly-developed arterial enhancement in the non-enhancing portion of the original mass or new lesions that satisfied the AASLD criteria for HCC^3^ appearing on subsequent follow-up imaging.

### Statistical analysis

To compare variables between the CR and non-CR groups, categorical variables (including MR imaging findings) were analyzed using the chi-square or Fisher’s exact test. Continuous variables were evaluated using the Mann–Whitney *U*-test. A logistic regression analysis was performed to identify the predictive factors for CR with DEB-TACE. Variables with a *p* value < 0.05 in the univariate logistic regression analysis were entered into the multivariate logistic regression analysis to determine the independent predictors. Odds ratios (ORs) with 95% confidence intervals (CIs) were estimated. The Cox proportional hazard model was used to identify prognostic factors associated with DFS in the CR group. DFS was defined as the interval between the administration of DEB-TACE and follow-up CT imaging that indicated recurrence. Multivariate models were created using variables that were significant in the univariate analysis (*p* < 0.05). Hazard ratios (HRs) with 95% CIs were calculated for each factor. All statistical analyses were performed using a statistical software package (SPSS version 25.0, IBM Corp.), and significance was defined as *p* < 0.05.

## Results

The patients’ baseline characteristics are summarized in Table 2. Fifty-five patients were evaluated in our study. Among them, 27 patients achieved CR, 15 achieved PR, and the others showed SD after the first session of DEB-TACE. Forty-three patients (78.2%) were classified as Child–Pugh class A, one patient from the non-CR group was classified as Child–Pugh class C, and the others were considered Child–Pugh class B. There were no significant differences between the two groups in terms of age, sex, etiology of liver disease, presence of liver cirrhosis, Child–Pugh class, or laboratory findings involving AFP and PIVKA-II (*p* > 0.05). DEB particles of 70–150 μm were used for 33 patients (60.0%) and that of 100–300 μm or 300–500 μm were used for others. But the size of DEB particles was not associated with the difference of the treatment response between the CR and non-CR groups (66.7% *vs.* 53.6%; *p* = 0.322).

**Table 2:** Baseline patient characteristics.

Characteristics	CR (n = 27)	Non-CR (n = 28)	*p* value
Age (years)	67.96 ± 11.57	65.71 ± 12.31	0.704
**Sex**			
Male	21 (77.8)	21 (75.0)	0.808
Female	6 (22.2)	7 (25.0)	
**Etiology of liver disease**			
Viral	16 (59.3)	19 (67.9)	0.508
Non-viral	11 (40.7)	9 (32.1)	
**Liver cirrhosis**			
Absent	5 (18.5)	6 (21.4)	0.787
Present	22 (81.5)	22 (78.6)	
**Child–Pugh class**			
A	21 (77.8)	22 (78.6)	0.943
B or C	6 (22.2)	6 (21.4)	
Albumin (g/dL)	4.1 (2.6–4.7)	3.8 (2.7–5.1)	0.448
Total bilirubin (mg/dL)	0.6 (0.3–1.9)	0.8 (0.3–2.4)	0.427
Prothrombin time (INR)	1.15 (0.97–1.94)	1.12 (0.99–1.70)	0.844
**AFP (ng/mL)**			0.777
< 30	12 (44.4)	14 (50.0)	
≥ 30	14 (51.9)	14 (50.0)	
NA	1	0	
**PIVKA-II (mAU/mL)**			
< 100	14 (51.9)	14 (50.0)	0.662
≥ 100	11 (40.7)	14 (50.0)	
NA	2	0	

Data are presented as mean ± SD, number (%), or median (range).

*AFP* alpha-fetoprotein, *CR* complete response, *INR* international normalized ratio, *NA* not available, *PIVKA-II* protein induced by vitamin K absence or antagonist-II.

Regarding MR imaging features, tumor size was significantly different between the groups. The proportion of patients with a tumor size ≥ 5 cm was smaller in the CR group than in the non-CR group (14.8% *vs.* 42.9%; *p* = 0.037). The median tumor size of all the included patients was 3.1 cm (range 1.3–8.5 cm). Additionally, heterogeneous arterial enhancement and heterogeneous signal intensity in the HBP were significantly greater in the non-CR group than in the CR group (55.6% *vs.* 82.1%; *p* = 0.033 and 18.5% *vs.* 60.7%; *p* = 0.001, respectively). Eighteen patients had multiple lesions: nine had two lesions, five had three lesions, and the others had four or more lesions. The proportion of patients with the central location was larger in the non-CR group. However, the multiplicity and location of the tumors did not affect the treatment response (25.9% *vs.* 39.3%; *p* = 0.291 and 11.1% *vs.* 32.1%; *p* = 0.101, respectively). All the other MRI findings also were not significantly different between the two groups (Table 3).

**Table 3:** MRI features of target lesions.

Characteristics	CR (n = 27)	Non-CR (n = 28)	*p* value
**Tumor size (cm)**			0.037
< 5	23 (85.2)	16 (57.1)	
≥ 5	4 (14.8)	12 (42.9)	
**Tumor number**			0.291
Single	20 (74.1)	17 (60.7)	
Multiple	7 (25.9)	11 (39.3)	
**Tumor location**			0.101
Peripheral	24 (88.9)	19 (67.9)	
Central	3 (11.1)	9 (32.1)	
**Heterogeneity on AP**			0.033
Homogeneous	12 (44.4)	5 (17.9)	
Heterogeneous	15 (55.6)	23 (82.1)	
**Rim enhancement on AP**			0.205
Absent	19 (70.4)	24 (85.7)	
Present	8 (29.6)	4 (14.3)	
**Peritumoral enhancement on AP**			0.702
Absent	18 (66.7)	20 (71.4)	
Present	9 (33.3)	8 (28.6)	
**Radiologic capsule**			0.937
Absent	20 (74.1)	21 (75.0)	
Present	7 (25.9)	7 (25.0)	
**Marginal irregularity**			0.349
Absent	14 (51.9)	11 (39.3)	
Present	13 (48.1)	17 (60.7)	> 0.999
**Peritumoral low SI on HBP**			
Absent	23 (85.2)	23 (82.1)	
Present	4 (14.8)	5 (17.9)	
**Heterogeneity on HBP**			0.001
Homogeneous	22 (81.5)	11 (39.3)	
Heterogeneous	5 (18.5)	17 (60.7)	
**Gross vascular invasion**			0.611
Absent	26 (96.3)	25 (89.3)	
Present	1 (3.7)	3 (10.7)	
**Conspicuous rim**			0.660
Absent	17 (63.0)	16 (57.1)	
Present	10 (37.0)	12 (42.9)	
**Peritumoral high SI on T2WI**			0.503
Absent	21 (77.8)	24 (85.7)	
Present	6 (22.2)	4 (14.3)	
**Intratumoral fat**			0.246
Absent	22 (81.5)	19 (67.9)	
Present	5 (18.5)	9 (32.1)	
**Intratumoral high SI on T1WI**			0.589
Absent	21 (77.8)	20 (71.4)	
Present	6 (22.2)	8 (28.6)	

*AP* arterial phase, *CR* complete response, *HBP* hepatobiliary phase, *SI* signal intensity, *T1WI* T1-weighted image, *T2WI* T2-weighted image.

**Figure 2:**
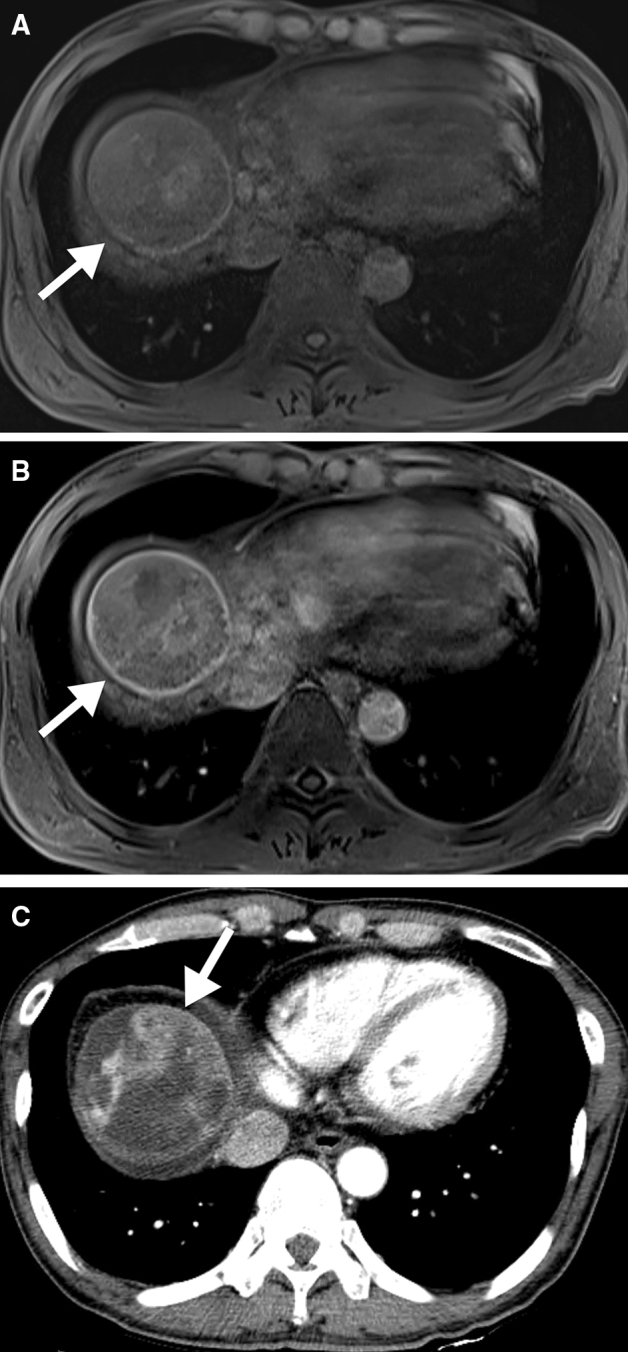
A 50-year-old man with hepatocellular carcinoma in hepatic dome (arrows). Gadoxetic acid-enhanced MR images show hypointensity in the precontrast scan **(A)** and heterogeneous signal intensity in the hepatobiliary phase **(B)**. Residual tumor with arterial enhancement remains on a follow-up CT taken 27 days after DEB-TACE **(C)**. Treatment response was determined to be a stable disease. *DEB-TACE* drug eluting bead transarterial chemoembolization, *CT* computed tomography, *MR* magnetic resonance.

Logistic regression was performed to determine the predictive factors of non-CR. In the univariate analysis, tumor size, signal heterogeneity in the arterial phase, and signal heterogeneity in the HBP were significantly different between the CR and non-CR groups (Fig. 2). However, in the multivariate analysis, only heterogeneous signal intensity in the HBP was significantly different between the groups (OR = 4.807; 95% CI = 1.011–22.865; *p* = 0.048) (Table 4).

**Table 4:** Logistic regression analysis of predictive factors for non-complete response.

Variable	Univariate analysis			Multivariate analysis		
	OR	95% CI	*p* value	OR	95% CI	*p* value
Age	0.984	0.940–1.029	0.481			
Sex (male)	0.857	0.246–2.983	0.809			
Etiology (viral)	1.451	0.481–4.377	0.508			
Liver cirrhosis	0.833	0.221–3.138	0.788			
Child–Pugh class (B or C)	0.955	0.265–3.433	0.943			
AFP (≥ 30 ng/mL)	0.857	0.294–2.497	0.778			
PIVKA-II (≥ 100 mAU/mL)	1.273	0.431–3.758	0.662			
Tumor size (≥ 5 cm)	4.312	1.176–15.809	0.027	1.286	0.233–7.081	0.773
Multiple tumors	1.849	0.587–5.822	0.294			
Central tumor location	3.789	0.899–15.972	0.070			
Heterogeneity on AP	3.680	1.076–12.583	0.038	1.663	0.401–6.888	0.483
Rim enhancement on AP	0.396	0.103–1.516	0.176			
Peritumoral enhancement on AP	0.800	0.254–2.516	0.703			
Radiologic capsule	0.952	0.283–3.205	0.937			
Marginal irregularity	1.664	0.571–4.853	0.351			
Peritumoral low SI on HBP	1.250	0.297–5.256	0.761			
Heterogeneity on HBP	6.800	1.983–23.314	0.002	4.807	1.011–22.865	0.048
Gross vascular invasion	3.120	0.304–32.029	0.338			
Conspicuous rim	1.275	0.432–3.761	0.660			
Peritumoral high SI on T2WI	0.583	0.145–2.352	0.449			
Intratumoral fat	2.084	0.595–7.302	0.251			
Intratumoral high SI on T1WI	1.400	0.412–4.756	0.590			

*AFP* alpha-fetoprotein, *AP* arterial phase, *CI* confidence interval, *HBP* hepatobiliary phase, *OR* odds ratio, *PIVKA-II* protein induced by vitamin K absence or antagonist-II, *SI* signal intensity, *T1WI* T1-weighted image, *T2WI* T2-weighted image.

Recurrent lesions were detected in 19 of the 27 patients with CR (70.4%), with a median DFS of 12 months (range, 3–29 months). The Cox proportional hazard model was used to evaluate the DFS after treatment. In the univariate analysis, AFP and peritumoral low signal intensity in the HBP were significant factors related to DFS. After including these variables in the multivariate analysis, the only significant parameter was elevated serum AFP level (≥ 30 ng/mL) (HR = 2.916; 95% CI 1.048–8.108; *p* = 0.040) (Table 5).

**Table 5:** Predictive factors for disease-free survival after complete response.

Variable	Univariate analysis			Multivariate analysis		
	HR	95% CI	*p* value	HR	95% CI	*p* value
Sex (male)	0.927	0.306–2.809	0.893			
Etiology (viral)	2.324	0.833–6.485	0.107			
Child–Pugh class (B or C)	1.086	0.384–3.069	0.131			
AFP (≥ 30 ng/mL)	3.415	1.293–9.016	0.013	2.916	1.048–8.108	0.040
PIVKA-II (≥ 100 mAU/mL)	1.308	0.513–3.336	0.574			
Tumor size (≥ 5 cm)	0.507	0.116–2.206	0.365			
Multiple tumors	2.173	0.811–5.824	0.123			
Central tumor location	0.251	0.033–1.894	0.180			
Heterogeneity on AP	0.789	0.319–1.950	0.608			
Rim enhancement on AP	0.843	0.302–2.349	0.744			
Peritumoral enhancement on AP	0.866	0.328–2.288	0.771			
Radiologic capsule	0.706	0.234–2.133	0.537			
Marginal irregularity	1.891	0.752–4.756	0.176			
Peritumoral low SI on HBP	4.143	1.236–13.887	0.021	2.488	0.711–8.710	0.154
Heterogeneity on HBP	0.158	0.021–1.200	0.075			
Gross vascular invasion	4.700	0.548–40.281	0.158			
Conspicuous rim	0.818	0.309–2.166	0.686			
Peritumoral high SI on T2WI	1.698	0.609–4.736	0.312			
Intratumoral fat	0.902	0.298–2.729	0.855			
Intratumoral high SI on T1WI	0.620	0.180–2.139	0.450			

*AFP* alpha-fetoprotein, *AP* arterial phase, *CI* confidence interval, *HBP* hepatobiliary phase, *HR* hazard ratio, *PIVKA-II* protein induced by vitamin K absence or antagonist-II, *SI* signal intensity, *T1WI* T1-weighted image, *T2WI* T2-weighted image.

## Discussion

In this study, we assessed potential factors for predicting treatment response and outcomes in HCC patients who undergo DEB-TACE. The only independent predisposing factor for non-CR was heterogeneous signal intensity in the HBP, with generally better treatment response for tumors showing homogeneous signal intensity in the HBP. Tumor size and heterogeneous arterial enhancement were significant factors in the univariate analysis but were not independent predictive factors in the multivariate analysis. AFP and peritumoral low signal intensity in the HBP were also significant factors related to DFS in the univariate analysis; however, only elevated serum AFP was an independent prognostic factor associated with shortened DFS in the CR group.

The degree of signal intensity in the HBP using GA-enhanced MR imaging is determined by organic anion-transporting polypeptide 8 (OATP8), since it is responsible for the intracellular uptake of GA^24,26^. Several studies have noted the role of OATP8 as a drug uptake transporter^27,28^. These studies suggest that OATP8 is involved in the influx of doxorubicin, so HCC with a high expression of OATP8 is likely to respond well to TACE. However, if a single tumor shows heterogeneous signal intensity in the HBP, it means that there are various degrees of tumor differentiation and OATP8 levels coexisting inside the tumor. HCC is well-known to be heterogeneous morphologically and genetically. Heterogeneous signal intensity in the HBP suggests the tumoral heterogeneity, which is associated with drug resistance in HCC and may influence treatment outcomes^29^. Our study showed that heterogeneous signal intensity in the HBP was a predictive factor of poor treatment response in the multivariate analysis.

Fujita et al.^30^ demonstrated that heterogeneous signal intensity in the HBP was associated with worse disease-free survival after surgical resection than homogeneous hypointensity in the HBP. However, Bae et al.^16^ suggested that heterogeneous signal intensity in the HBP was not associated with the prognosis after surgical resection, RFA, or conventional TACE, which seems to contradict previous findings. In our study, heterogeneous signal intensity in the HBP was not an independent factor for shortened DFS after CR.

Heterogeneous arterial enhancement has been associated with worse treatment response and poor prognosis in conventional TACE and DEB-TACE^19,31,32^. Tumor size is also a well-known predictor related to response and prognosis for conventional TACE^12,15,17,18,33–35^ and DEB-TACE^20,31^. These results can be explained by the degree of tumor differentiation, as a poorly differentiated HCC is more likely to manifest in larger tumor size and heterogeneous arterial enhancement^36,37^. These factors are related to the lower arterial blood supply, which leads to decreased tumor staining during the TACE procedure^18,19,38^. Therefore, doxorubicin is not evenly distributed inside these tumors, reducing the intratumoral drug concentration, and decreasing the therapeutic effect of TACE. Poorly differentiated HCC has been shown to respond poorly to DEB-TACE^6^. Our study showed that heterogeneous arterial enhancement and larger tumor size were significant in the univariate analysis, but not independent risk factors of treatment response in the multivariate analysis. Further investigations are needed to determine the correlations among tumor size, heterogeneous arterial enhancement, and treatment outcomes.

Peritumoral hypointensity in the HBP has already been reported as a major factor related to microvascular invasion^16,17^. Furthermore, previous studies have suggested that microvascular invasion is a predictor of early recurrence after surgical resection of HCC^23,39^. One possible explanation for this is the change in peritumoral perfusion resulting from microvascular invasion, which may influence OATPs, reducing the uptake of GA in hepatocytes around the tumor^23^. Two recent studies have investigated the predictive value of peritumoral hypointensity in the HBP for response to conventional TACE and survival, but they showed contradictory results^16,17^. In our study, peritumoral hypointensity in the HBP was not an independent factor for shortened DFS. Therefore, further studies on peritumoral hypointensity in the HBP and its effect on the prognosis of patients with HCC after DEB-TACE are necessary.

High serum AFP has been found to be a risk factor for incomplete response, early recurrence, and poor outcomes after TACE^32–35,40^. However, only one of these studies focused on DEB-TACE specifically^40^, and a multivariate analysis was not performed in that study. Generally, high levels of serum AFP in HCC are associated with a worse differentiation grade than low levels of serum AFP. In addition, decreased serum AFP levels after TACE is considered a positive response to treatment and is also used as a predictor of prognosis^34^. In our multivariate analysis, we found that elevated serum AFP (≥ 30 ng/mL) was the only independent factor predicting shorter DFS after CR.

There were several limitations to our study. First, it was a retrospective study design of a single center. Second, the patient population was relatively small. However, to our knowledge, this is the first study about the correlation of pretreatment GA-enhanced MRI findings with DEB-TACE results. A study including a larger number of patients would be necessary for further validation of these results. Third, there was a discrepancy in the size of DEB particles between the patients, which may be a confounding factor since smaller DEB particles were shown to have better efficacy in some previous studies^20,21^. However, in our study, the particle size was not significant factor for treatment response.

In conclusion, heterogeneous signal intensity in the HBP on GA-enhanced MRI may be a helpful biomarker to predict non-CR after DEB-TACE in patients with HCC. And elevated serum AFP was a valuable factor for predicting shorter DFS after DEB-TACE.
